# Beyond the target insects: impacts of Bti on aquatic macrofauna communities

**DOI:** 10.1186/s13071-025-06907-8

**Published:** 2025-07-09

**Authors:** Julia C. van Nieuwpoort, Maarten Schrama, Jeroen Spitzen, Sam Philip Boerlijst

**Affiliations:** 1 Institute for Environmental Sciences Leiden, Department of Environmental Biology, University of Leiden, Einsteinweg 2, 2333CC Leiden, Netherlands; 2 Naturalis Biodiversity Center, P.O. Box 9517, 2300 RA Leiden, Netherlands; 3 Netherlands Institute for Vectors, Invasive Plants and Plant Health (NIVIP), Centre for Monitoring of Vectors (CMV), Netherlands Food and Consumer Product Safety Authority (NVWA), Wageningen, Netherlands; 4 Deltares, Division of Inland Water Systems, P.O. Box 177, 2600 MH Delft, Netherlands

**Keywords:** Ecotoxicology, Non-target effects, Trophic interactions, Biological larvicides, Freshwater ecosystems, Aquatic invertebrates

## Abstract

**Background:**

The larvicide *Bacillus thuringiensis israelensis* (Bti) was introduced as a pest control method in the 1980s, claiming not only to be effective, but also to target specific insect groups including Culicidae and Simulidae, with no substantial effects on non-target species or to the local ecosystem.

**Methods:**

To test these claims, we applied Bti to a naturally colonized, replicated set of aquatic macrocosms under realistic, field-like conditions, and investigated impacts on non-target species, including effects on related taxa and higher trophic levels.

**Results:**

Our results indicate limited effects on most invertebrate prey taxa, with the exception of a decimation of the Chironomidae, which compose up to 50% of the biomass of these aquatic ecosystems. Effects on invertebrate predators including Hemiptera, Odonata and Coleoptera were investigated but were only observed for 1 species of Odonata: a small but significantly negative effect on larvae of *Ishnura elegans* (Odonata: Zygoptera).

**Conclusions:**

Overall, our results support the claim that, when used during short intervals at small spatial extents, Bti has limited effects on aquatic ecosystems, and that effects are relatively short-lived. However, negative effects on Chironomidae and Odonata larvae warrant careful use of the substance at natural water ecosystems, especially as the former insect family constitutes the basis of the aquatic food in soft-sediment freshwater aquatic systems and Bti leads to a local temporary near-complete wipeout of this Diptera family. Overall, our results highlight the need to define and limit the spatial and adhere to the advised temporal extent at which Bti is used.

**Graphical Abstract:**

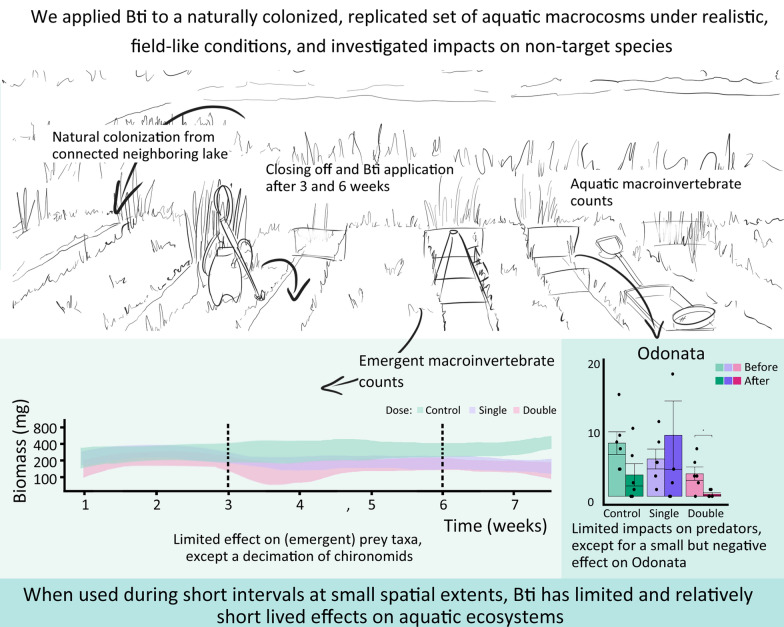

**Supplementary Information:**

The online version contains supplementary material available at 10.1186/s13071-025-06907-8.

## Background

To limit public health risk resulting from mosquito-borne pathogens, various control methods have been proposed to suppress mosquito populations over the past decades. Chemical insecticides have proven to be quick and effective in the short term, but extensive and continuous use of these biocides has induced resistance in mosquitoes [1], decreasing their effectiveness. In addition, chemical insecticides can have broad-spectrum effects harming non-target species and disrupting ecosystems over time [2], leading to the loss of ecosystem services such as pollination and soil regulation [3]. Further bioaccumulation of chemicals can lead to adverse effects on human health. Therefore, the use of chemical insecticides has been heavily regulated under EU Biocidal Products Regulation (BPR, Regulation (EU) 528/2012) as well as many national laws [4]. Consequently, various active substances that have been used in the past, have been banned in Europe [5].

The bacterium *Bacillus thuringiensis israelensis* (Bti) was introduced as a biologically friendly alternative active substance for biocides in the 1980s. Products based on Bti are commonly used as a target-specific measure, in aquatic systems targeting larvae of Diptera aimed specifically at Culicidae and Simuliidae [6], but also targeting other closely related Diptera such as chironomids. The uptake by non-target species is minimized by adapting to filter feeding behaviours of mosquito larvae, which ‘drink’ free nutrients in the water. Upon uptake, the active bacteria release crystalline proteins (protoxins) during sporulation. These protoxins are dissolved in the midgut of the mosquito larvae under the low pH environment. Through this process, delta-endotoxins are formed, composed of Cry and Cyt toxins which bind to specific Bti receptors present in target organisms. This leads to perforation in the midgut. Consequently, larval death will occur within 2–24 h as a result of starvation [7]. This target specificity implies that it should not harm insects with a different feeding mode and phylogenetically unrelated species, such as species that predate on the target species or share their habitat [8]. This specificity is determined by the mode of action of Bti [7]. Moreover, Bti is claimed to be environmentally safe, has been widely commercially available since the 1980 s [9], and does not have negative effects on bacterial and algal composition, as well as crops [10]. Moreover, its toxins are described as completely biodegradable [11], as well as its spores, because of which the concentration of spores is not expected to exceed normal background levels of *Bacillus thuringiensis* [12]. However, concerns have been raised as to the implications of the limited persistence for environmental safety and resistance acquisition [4, 13, 14].

Due to the widening concern of global decline of biodiversity, this paper will re-examine a number of concerns regarding its widespread use. First, aside from the aquatic target organisms Culicidae and Simuliidae, other phylogenetically close taxa in aquatic ecosystems, such as Chironomidae, are suspected to ingest Bti via the same uptake mechanism: filter feeding. Chironomidae are often the most abundant invertebrate group in freshwater ecosystems [15] and can account for up to 50% of the animal biomass in freshwater ditches in Northwest Europe [16]. Previous studies show conflicting results regarding the Bti susceptibility of Chironomidae. This may be due to variation in feeding behaviour; not all chironomid species share the filter-feeding behaviour of Culicidae [17, 18]. The second concern is that a decline in Dipteran larvae, such as the abovementioned Chironomidae, could affect higher trophic levels inside aquatic systems (e.g. insect orders Hemiptera, Coleoptera, Odonata) and in the adjacent terrestrial systems due to emerging insects and their bridging function between aquatic and terrestrial food webs [19]. In various wetland systems Bti has been shown to have negative effects on a variety of species in different trophic levels. Östman, Lundström, and Vinnersten showed that protozoan densities in Bti-treated natural wetlands were on average 4.5 times higher than the control because of the loss of filter feeding Diptera larvae [20]. Additionally, the fitness of insectivorous birds declined due to the loss of Nematoceran prey and their predators [21]. Similarly, the survival rates of frog and newt larvae have previously been reported to be reduced presumably due to a shift in predator–prey relations in the presence of Bti [8, 22]. Overall, the abovementioned concerns suggest that the flow of energy and trophic interactions within an ecosystem might be altered through the indirect effects of Bti applications, but particularly quantitative literature on the ensuing effects on trophic interactions in natural aquatic systems remains scarce.

Hence, the aim of this study is twofold. Firstly, to assess and quantify the extent and longevity of direct effects on phylogenetically close taxa among the non-target insect communities, with a particular focus on the Chironomidae. Secondly, to determine potential impacts of changes in food availability and subsequent alterations to the foodweb on higher trophic levels. We expect that all chironomids may be affected by Bti, but that the extent depends on the feeding guild and subsequent exposure. As chironomids constitute a large portion of (emergent) biomass in freshwater systems, we expect that prey-switching will occur for predator species. To this end, we have set up a macrocosm experiment at the Living Lab in Leiden, The Netherlands, to assess the effects of Bti in a naturally assembled ecosystem with low densities of Culicinae and Anophelinae [16, 23].

## Methods

### Experimental set-up

To study the various impacts of Bti on freshwater non-target and predator communities, we set up a macrocosm experiment at the Living Lab field station in Leiden, the Netherlands [16], between April and June 2022. This study made use of 24 experimental freshwater ditches, which were constructed in 2017 (6 × 1 × 0.5 m), which mimic the natural conditions of the local ecosystem, and have soft sediments, but can be closed off from the connecting water body [6]. Four weeks prior to Bti application, the ditches were connected to the natural lake to allow for the colonization of the experimental ditches [16, 23]. To ensure that colonization led to similar communities, a null measurement of the local community was collected at *t* = 1 (week 1), and additionally at *t* = 2 (week 2); see below.

### Treatment application

At the end of the 3rd week (*t* = 3) and the start of the 6th week (*t* = 6) Bti was applied. Concentrations of Bti used were 0 (control), the highest recommended dose in the Netherlands (1000 g/ha; single) and twice the recommended dose (2000 g/ha; double) [12]. Each of the treatments had six replicates, for a total of 18 ditches. To ensure maximum realism, we harmonized the mode of application and reapplication of Bti with protocols used by professional pest control and provided by Centre for Monitoring Vectors (personal comm. NVWA/RIVM; Rentokil/KAD). In short, this entails the following procedure: 6 mg of VectoBac WG (Valent BioSciences; Libertyville; 3000 ITU/mg) was suspended in 1 L of dechlorinated tap water, homogenized and subsequently applied to the surface of the ditches using a high-pressure sprayer using 0.0104 L/m^2^. Reapplication was performed 2 weeks after according to the same protocol. To monitor and potentially correct for confounding effects, several abiotic measurements were taken on a weekly basis. These included nutrient levels (in the form of phosphorus and nitrogen), water temperature, dissolved oxygen content, algae concentrations, and turbidity.

### Direct effects on non-target insects

To ensure a comparable estimation of the number and composition of insect specimen emerged in the aquatic system, each ditch was equipped with emergence traps measuring 0.36m^2^ (60 × 60 cm), based on Barmentlo et al. [16]. In short, emergence traps were placed slightly below the water surface level and secured with tent pegs to prevent emerging insects from escaping regardless of water level fluctuations and to provide Odonata the opportunity to climb the nets for pupation. Polyethylene bottles containing 70% ethanol were attached to the traps to trap and preserve the insects. Samples from the emergence traps were collected on a weekly basis, and the preservation medium was changed in parallel. Trapped insects in the emergence traps were preserved in 50 ml falcon tubes containing ethanol (70%) awaiting morphological identification congruent with the methods of Barmentlo [16]. Samples were subsequently identified to subfamily level. Length was measured for to 5 individuals per species (depending on availability). Hereafter, biomass was determined per order, family, feeding guild, and over the system as a whole following a length-dry weight power equation for adult insects [24].

### Effects on closely related taxa and higher trophic levels

To obtain a baseline of the species composition of the macroinvertebrate community prior to Bti applications, all ditches were sampled to examine the macroinvertebrate predator community, approximately one week prior to Bti application. For each of the 18 experimental ditches, 1 m was temporarily closed off using a metal plate. All macroinvertebrates were removed from this section by a net, followed by a 1 m diameter 300-µm sieve, after which the metal plates were removed again. All individuals were split by order, and thereafter identified to species and counted. The organisms and organic material were returned to the corresponding ditch. Approximately 2 weeks after the second round of Bti application, all ditches were sampled again for macroinvertebrates, using the method described above. Samples were subsequently divided into corresponding order, family, genus, species, and assigned to feeding guilds. Length was measured for to 5 individuals per species (depending on availability). Hereafter, biomass was determined per order, family, feeding guild, and over the system as a whole following a length-dry weight power equation for aquatic invertebrates [25].

### Data processing

All data was analysed using R v 4.3.2 [26]. To evaluate the emergence data and assess the direct effects of Bti on non-target species, we performed a Friedman test, using biomass per taxonomic level as dependent variable and sample date, ditch, treatment, and different groups as explanatory variables. Outliers were determined using the outlierTest function and assessed for their influence using the influenceIndexPlot function from the car package [27]. For all analyses, the same data points were identified as potential outliers. Three data points that were flagged as outliers turned out to be valid data points that contained insects that were comparatively large and therefore had high biomass. Whilst this could skew the results of the total biomass analysis over the system as a whole, these data were still left in considering these were no true outliers.

To test for possible differences in abiotic variables between treatments (PO₄, NO₃, water temperature, dissolved oxygen content and turbidity) and their potential interactions we ran a PCA and ANOSIM analysis using Bray–Curtis dissimilarity (Appendices A and B) using Past3 v1.0.0.0 [28]. These showed no clear variance and were thus excluded in subsequent analyses.

To test whether shifts in the composition of the ecosystem resulted from the application of Bti, we used generalized additive models with a zero-inflated Poisson distribution and logit link using gamlss [29]. These models compared the effect of treatment on guild, order, family, genus and species level using the data collected during macroinvertebrate sampling (Table 1). The models were optimized using backwards selection by AIC and used the fixed effect of groups and treatments, and the random effect of ditch. For comparative purposes, the reference group was set to a group that is commonly assumed to be unaffected by Bti [30]. Emergence data was analysed using Friedman tests. To get an overview of community-level differences, an Analysis of Similarities (ANOSIM) was used using Bray–Curtis dissimilarity (Appendix C). An in-depth analysis of orders potentially predating on mosquitoes was carried out, which included the following orders: Aranea, Coleoptera, Diptera, Hemiptera, Megaloptera, and Odonata.

**Table 1:** Generalized additive models used

Formula	Family	Global deviance	Sigma coefficients intercept			
			Estimate	Std. error	*t* value	Pr (>|t|)
Count ~ Guild * Treatment * Day + random (Ditch)	ZIP	1123.297	0.9132	0.1401	6.516	1.66e-10
Count ~ Order * Treatment * Day + random (Ditch)	ZIP	1984.052	0.8487	0.1023	8.293	1.07e-15
Count ~ Family + Treatment * Day + random (Ditch)	ZIP	3808.74	1.56059	0.06952	22.45	< 2e-16
Count ~ Genus + Treatment * Day + random (Ditch)	ZIP	4022.517	1.54475	0.06812	22.68	< 2e-16
Count ~ Species + Treatment * Day + random (Ditch)	ZIP	4096.186	1.81930	0.06613	27.51	< 2e-16

### Results and conclusions

We observed a total of 35 species across 5 orders (see appendix D for more info). Following application of Bti, we observed a number of clear effects on both target and non-target species, with a drop in total biomass of macrofauna of almost 50% for both Bti treatments (49.8% and 49.3% for single and double dose respectively) between application and the subsequent week (*P* = 0.04). Additionally, we observed a pronounced difference between the control and the double Bti dose in that same week (*P* = 0.03). By the end of the experiment, we once again observed a difference in biomass between the control group and both the single and double dose treatment (*P* = 0.03). Overall, the total biomass in the system seems to follow the trend: after Bti application, biomass decreases and then gradually recovers to its original level.

### Temporal effects

**Fig. 1:**
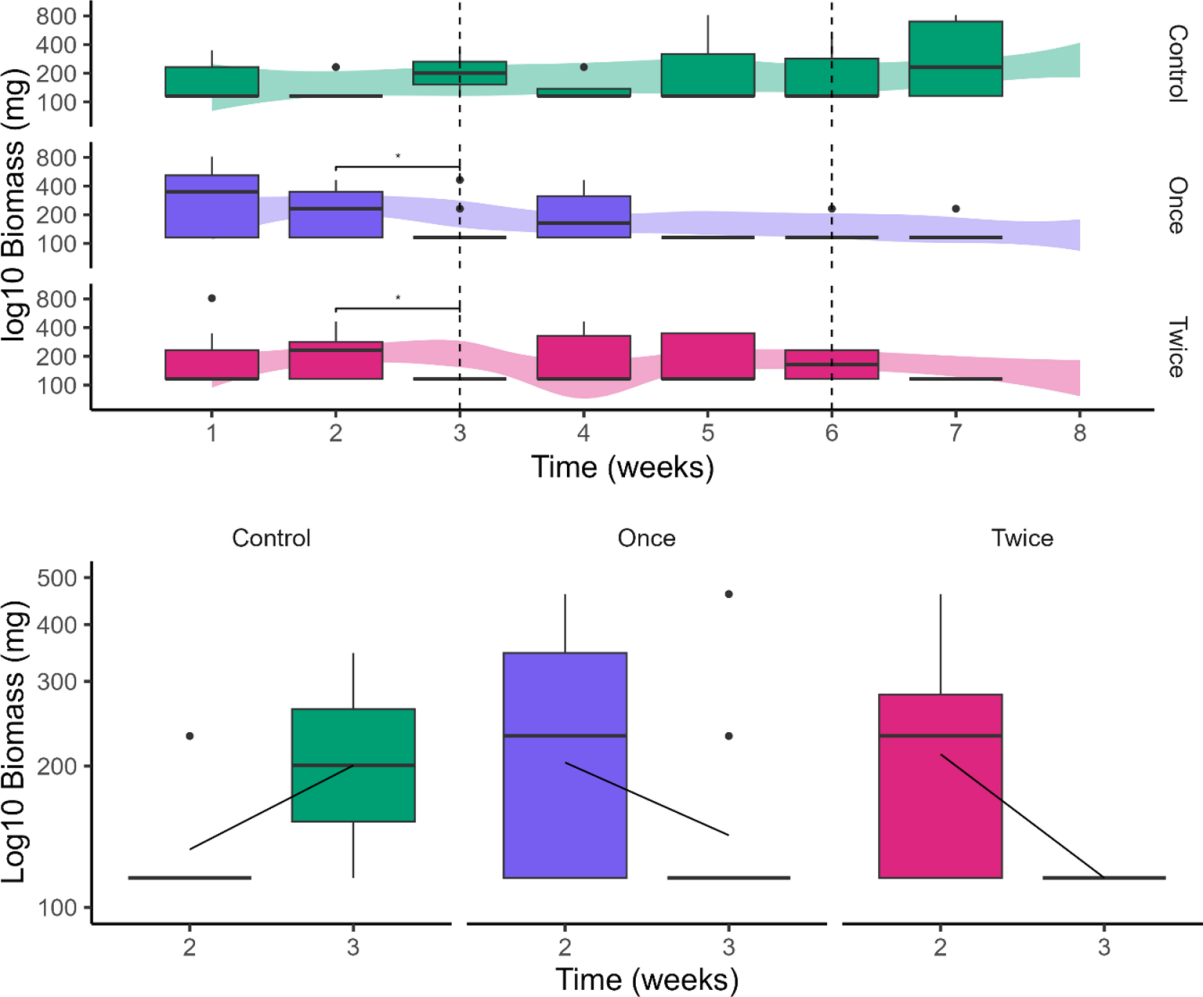
Biomass (log-transformed) of the Chironomidae family per meter ditch, plotted against the total biomass trend per treatment type, depicted using boxplots. The figure displays the overall trend observed throughout the entire experiment above underlayed by loess regression, with a focused excerpt showing the period just before and after the initial application below overlayed by the difference in mean as a black line

When examining the compositional shifts in emergent macroinvertebrates resulting from application of Bti, a clear pattern of decrease was observed at the order (Diptera) and family (Chironomidae) level. Considering the large majority of Diptera consists of Chironomidae, the analyses were carried out at the family level. Post hoc testing revealed the Chironomidae biomass to be lower after Bti application for the double treatment (*t* = 3, *P* = 0.006; 52% and 44% for first and second application respectively). The vast majority of individuals remaining belonged to the Tanypodinae (57% and 40% for single and double treatments respectively after the first application), a subfamily primarily consisting of predators [31–33]. Additionally, the biomass in both Bti treatments was lower than the control after the second round of Bti application (12.5% and 88% for single and double respectively), which is depicted in Fig. 1.

**Fig. 2:**
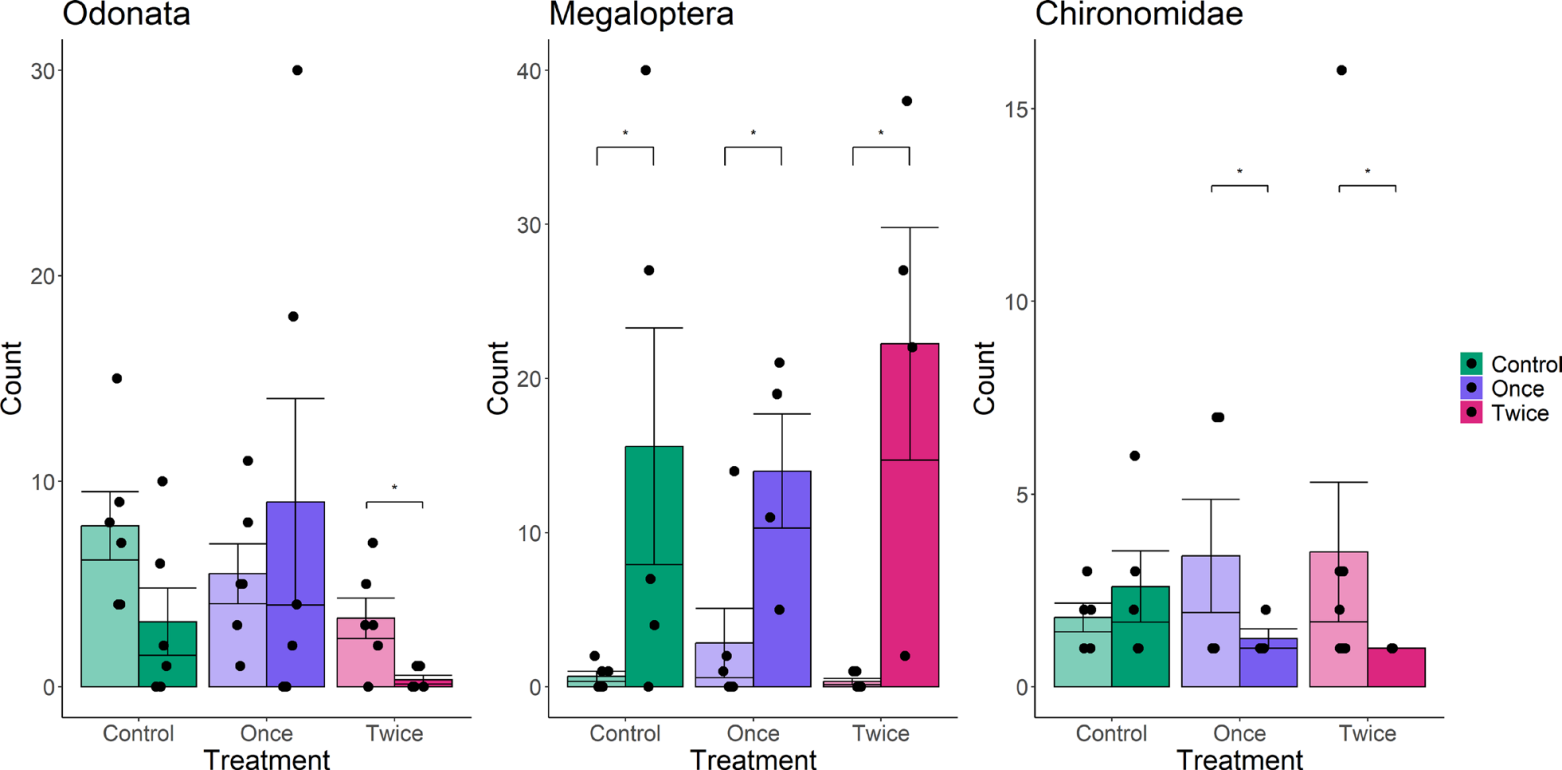
Abundance for the most responsive macroinvertebrate order (left two panels) and family (right panel), expressed as counts per meter of ditch for each treatment. The data are presented as bar charts with standard error, showing comparisons before (light) and after (dark) the application of Bti. Asterisks indicate significant differences before and after treatment application

For the predator data, the ANOSIM analysis showed no differences in community composition across the treatments (Bray–Curtis dissimilarity, *R* = 0, *P* > 0.05, stress = 0.14). This lack of significant variation was consistent across taxonomic levels, indicating no shift in community structure in response to Bti application. Despite the lack of significant differences at the community level, the Friedman analysis identified effects for two of the groups at specific treatments and measurement days. Megaloptera increased in abundance following the Bti application, but also in the control, therefore likely indicating a seasonal effect (Fig. 2). For Odonata, we observed a clear effect of Bti, but only in the treatment with the double dose, where the abundance was 79% lower after exposure (Fig. 2).

## Discussion

We applied Bti to a naturally assembled set of aquatic macrocosms. In line with our hypotheses, we found major, albeit short lived, effects on abundance and biomass of chironomids (non-biting midges), and limited effects on higher trophic levels. Effects of both the single and double dose of Bti were surprisingly short-lived; successful recolonization of water bodies was already observed within one week after application. These results reinforce the idea that at small temporal and spatial scales, application of Bti works if applied following its product label, but highlight the possible risks related to application at larger spatio-temporal scales in natural systems.

### Changes in species composition after Bti application

Previous studies have shown some conflicting results regarding the responses of aquatic taxa to application of Bti. Our study, carried out in well-replicated naturally colonized aquatic macrocosms, shows several clear effects. Clear negative effects were observed for the chironomids, which experienced a stark decline in numbers (> 50%) in the weeks immediately following application, largely in line with previous studies. Most of the chironomid specimen that survived belonged to non-filter feeding subfamily of Tanypodinae and have a primarily predatorial feeding mode [31–33], which may partly explain the contrasting results from previous studies. Within the Diptera, which constituted 68.1% of the total insect biomass in the control ditches, chironomids were the most abundant group (53.7% in the control ditches), which is an accurate reflection of Dutch aquatic ecosystems [34]. Hence, a steep drop in abundance of this group likely leads to a (albeit temporary) loss of detritus feeders and the functions they have in the ecosystem and might cause a (temporary) lack of food sources for predators that depend on them [35]. Related to this, our results showed a small but significant negative effect on larvae Ishnura elegans (Odonata: Zygoptera). There are three potential explanations for this Odonata-specific effect, which was not observed for any other aquatic predator. Firstly, there is the possibility of prey switching. Predators that typically target Chironomidae may have shifted to alternative prey sources [36]. Another possibility is that predator-predator interactions took place, with some groups feeding on the Odonata, which would explain why they were the only group affected. We know that Odonata can resort to intraguild predation, which is a common behaviour in these species but not for most other predator taxa [37]. Thirdly, the other predator species may have switched to scavenging. This implies that the predators that would usually predate on Chironomidae now scavenge on the dead larvae. Most invertebrate predator species are known to scavenge when conditions require them to do so [38]. Odonata are the exception to this, refusing to scavenge and preferring to hunt live prey [39]. This was confirmed in a small separate microcosm experiment, which showed a decrease of 40% in number of Bti-exposed larvae consumed compared to a control group (Appendix E). While all three explanations may be at play, the latter result might explain why the other predators survived, but Odonata experienced a drop.

### Recovery dynamics

While some insect orders showed a pronounced response to application of Bti, no medium-long effects were observed on the aquatic ecosystem as a whole. The observed rapid, and seemingly complete, recuperation of the aquatic macrofauna community within the 6-week timeframe of the experiment suggests that small freshwater systems such as used in this study may be able to bounce back quickly. It is important to emphasize that this experiment took place in a relatively small system with a buffer zone that allowed rapid recolonization. However, the recolonization potential of faunal communities likely depends on the scale at which Bti is applied. It is therefore likely that recuperation periods are longer in larger aquatic systems, or in systems where recurrent application takes place, but more work is needed to address this. In the meantime, caution is warranted about the safety of Bti based on the effects found in these results.

### Risks of expanding mosquito control

Both mosquito-borne pathogens as well as exotic invasive mosquito species are expected to spread to higher latitudes [40]. These increases may lead to further calls to suppress mosquito populations. To date, local introductions of *Aedes albopictus*, such as currently observed in the Netherlands and Flanders, are controlled both by larval source removal and the application of VectoBac WG or VectoMax FG. The latter larvicide differs from VectoBac with the addition of *Bacillus sphaericus*, which is effective over a longer period than treatment with Bti alone, and less prone to resistance. This type of usage is limited to small and isolated artificial breeding habitats, such as rain barrels and street gullies, likely with limited effects on other taxa [41]. This is in sharp contrast with expected results, if Bti were to be used to control species breeding in natural wetland ecosystems, such as *Culex pipiens*, *Aedes caspius* or *Aedes vexans*. In such ecosystems, where pronounced side effects on the total ecosystem can be expected according to the effects described above. Therefore, depending on the relation between the mosquito and its surrounding ecosystem, suppression with Bti can be expected to range from no significant harm to short term loss (weeks) of ecosystem function at the targeted water bodies.

## Conclusions

Overall, our results confirm the claim that, when used during short intervals at small spatial extents, Bti has limited effects on aquatic ecosystems, and these effects are relatively short-lived. However, the observed negative effects on Chironomidae and Odonata larvae warrant careful use of the active substance in natural water bodies. Chironomids constitute the basis of the aquatic food chain in soft-sediment freshwater aquatic systems and recurrent (and/or incorrect) use of Bti could lead to a local near-complete wipeout of this Diptera family. This therefore raises concerns for application at larger spatial scales or in isolated systems, as resulting limited recolonisation may make these systems particularly vulnerable. Similarly, as emerging insects impact both aquatic and terrestrial food webs, even relatively short disruptions should be considered for application in natural systems. For instance, timing in Bti application in relation to early life stages of univoltine species, may exacerbate or mitigate ecological impacts, underlining the importance of phenological context. Ultimately, this highlights the need to define and limit the spatial and temporal extent when Bti is used at natural water bodies.

## Supplementary Information

Additional file 1

## Data Availability

Data supporting the conclusions of this article are included within the article and its appendices (Additional file 1). The original datasets used and analysed during the present study are freely and openly available. All data generated or analysed during this study are included in this published article and Additional files 2–6.
