# Supplementary material for: Beyond the target insects: impacts of Bti on aquatic macrofauna communities
Source: Parasit Vectors. 2025 Jul 9;18:271. doi: 10.1186/s13071-025-06907-8 (PMC12239451; doi:10.1186/s13071-025-06907-8)
Supplement: Supplementary file 1 — Additional file 1 [file 13071_2025_6907_MOESM1_ESM.zip › Supplementary/Nieuwpoort et al appendix (2).docx]

**Appendices**

**Appendix A**

Principal Component Analysis Abiotic data

| Table 1A  *PCA Analysis Abiotic Data.* | | |
| --- | --- | --- |
| PC | Eigenvalue | % Variance |
| 1 | 2620.89 | 51.751 |
| 2 | 1613.89 | 31.868 |
| 3 | 813.488 | 16.063 |
| 4 | 10.5593 | 0.2085 |
| 5 | 4.74915 | 0.093775 |
| 6 | 0.78276 | 0.015456 |

*Note.* This table demonstrates the Principal Component Analysis for the abiotic measurements. PC 1 and 2 matched well with chlorophyll and turbidity, which explain 83% of variance.


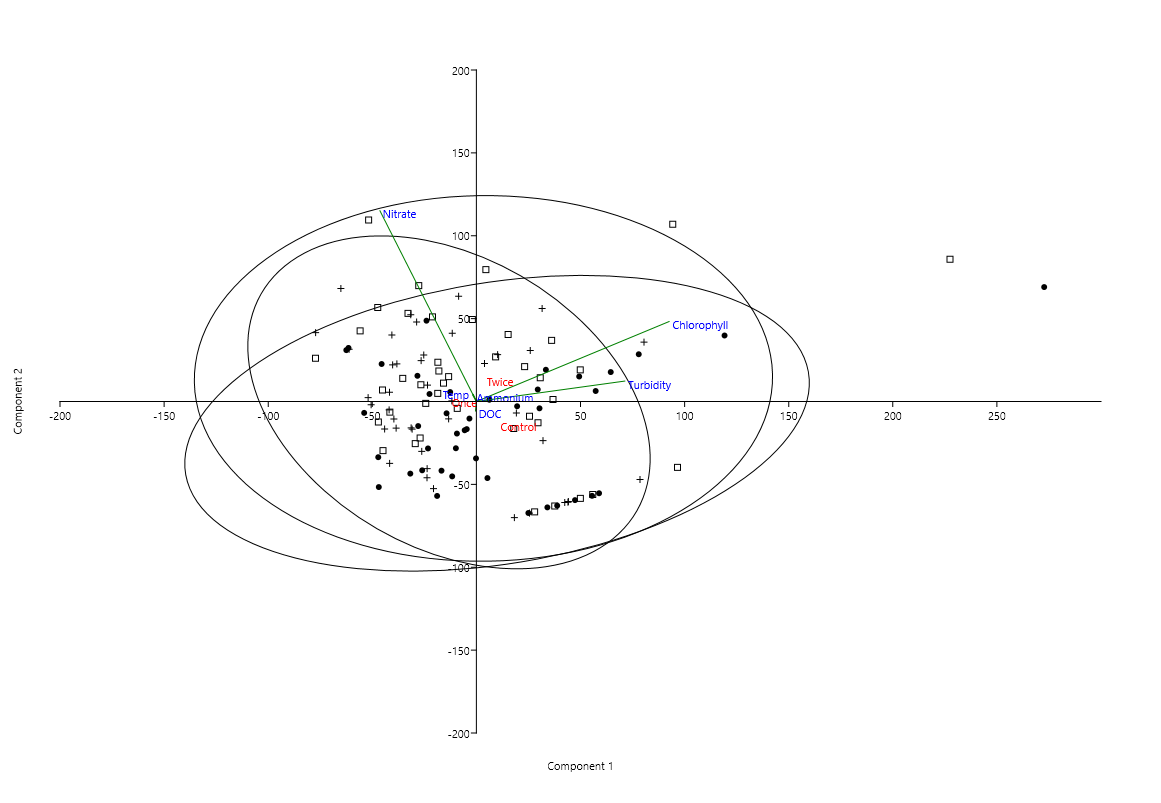


Fig. 1A Principal Component Analysis plot. PC 1 and 2 matched well with chlorophyll and turbidity, which explain 83% of variance.


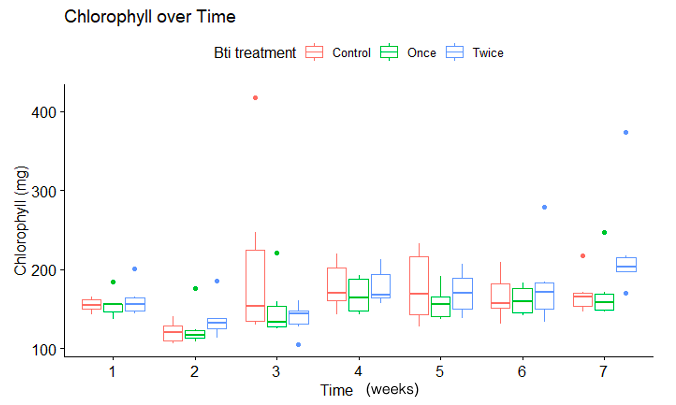


Fig. 2A Chlorophyll (mg) vs. Time (weeks). Since PC 1 and 2 aligned with chlorophyll and turbidity, this figure illustrates the chlorophyll in the system over time for the three Bti treatment groups.


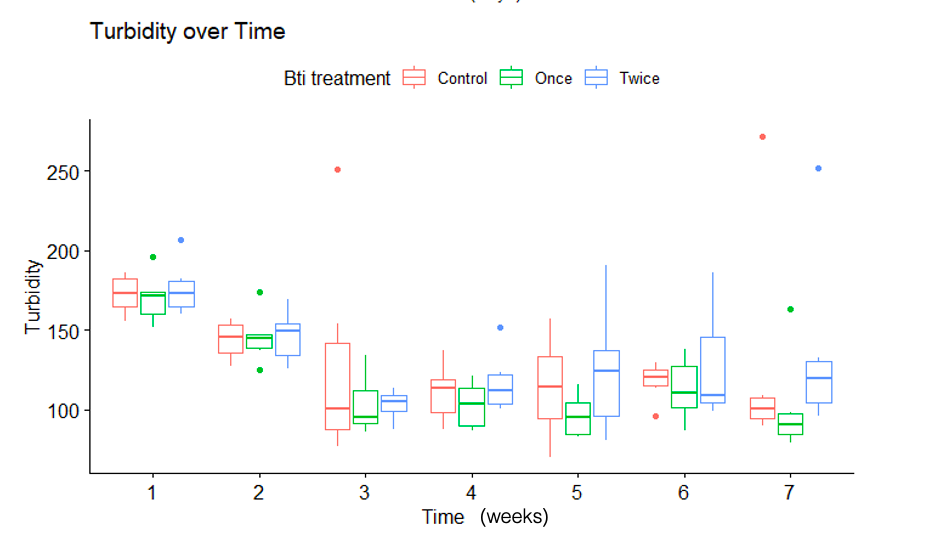


Fig. 3A Turbidity vs. Time (weeks). Since PC 1 and 2 aligned with chlorophyll and turbidity, this figure illustrates the turbidity levels in the system over time for the three Bti treatment groups.

**Appendix B**

Analysis of Similarities & Non-metric Multidimensional Scaling Abiotic data

| Table 1B  *Two-way ANOSIM Abiotic data.* | | |
| --- | --- | --- |
| Factor | R statistic | p-value |
| Treatment | 0.029005 | 0.1711 |
| Day | 0.45764 | 0.0001 |

*Note.* This table demonstrates the Non-metric Multidimensional Scaling Abiotic for the abiotic measurements.


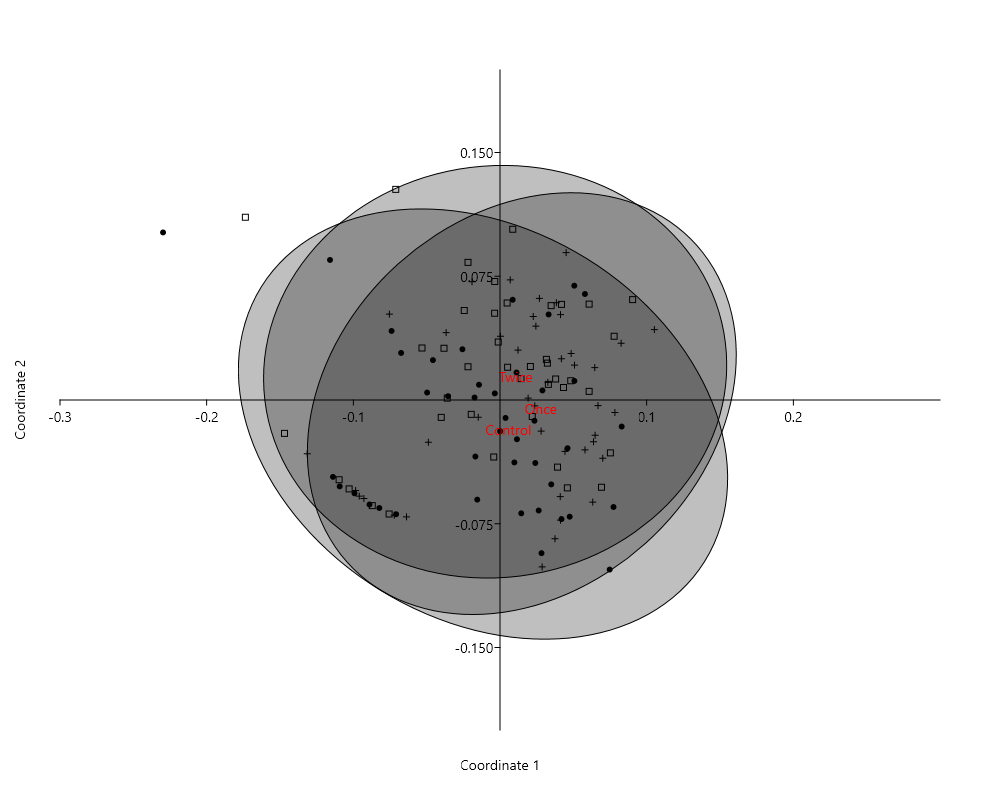


Fig. 1B nMDS ordination for Coordinate 2 vs. 1. Overall clustering shows no severe patterns. Stress = 0.08037.


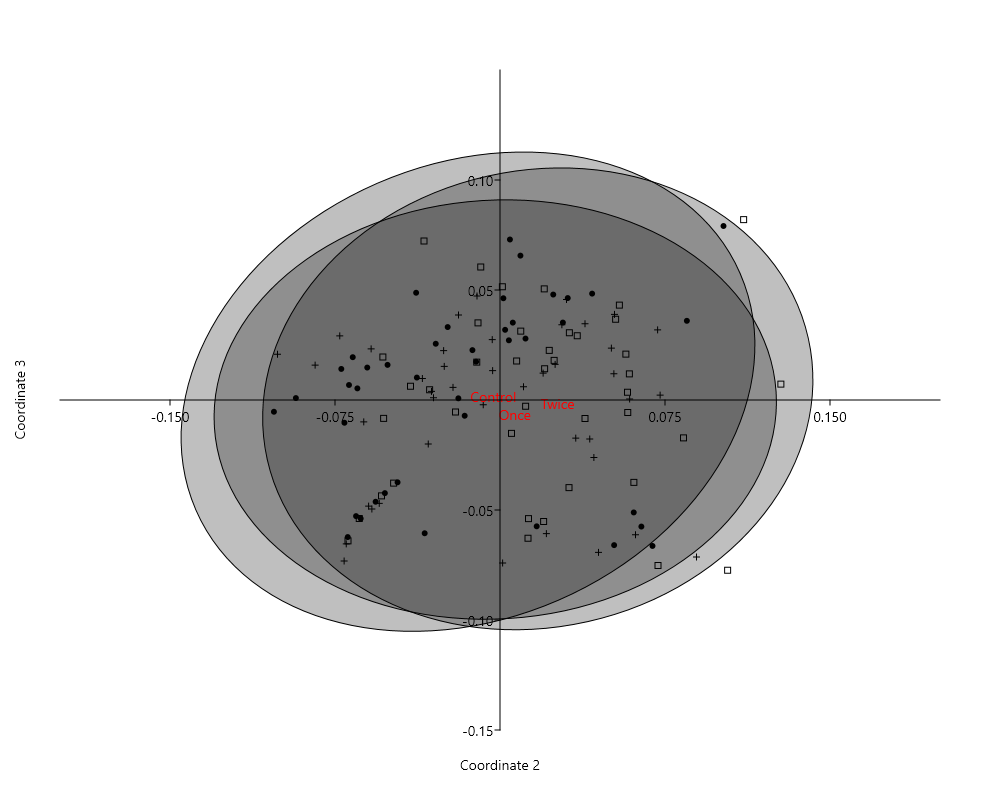


Fig. 2B nMDS ordination for Coordinate 3 vs. 2. Overall clustering shows no severe patterns. Stress = 0.08037.

**
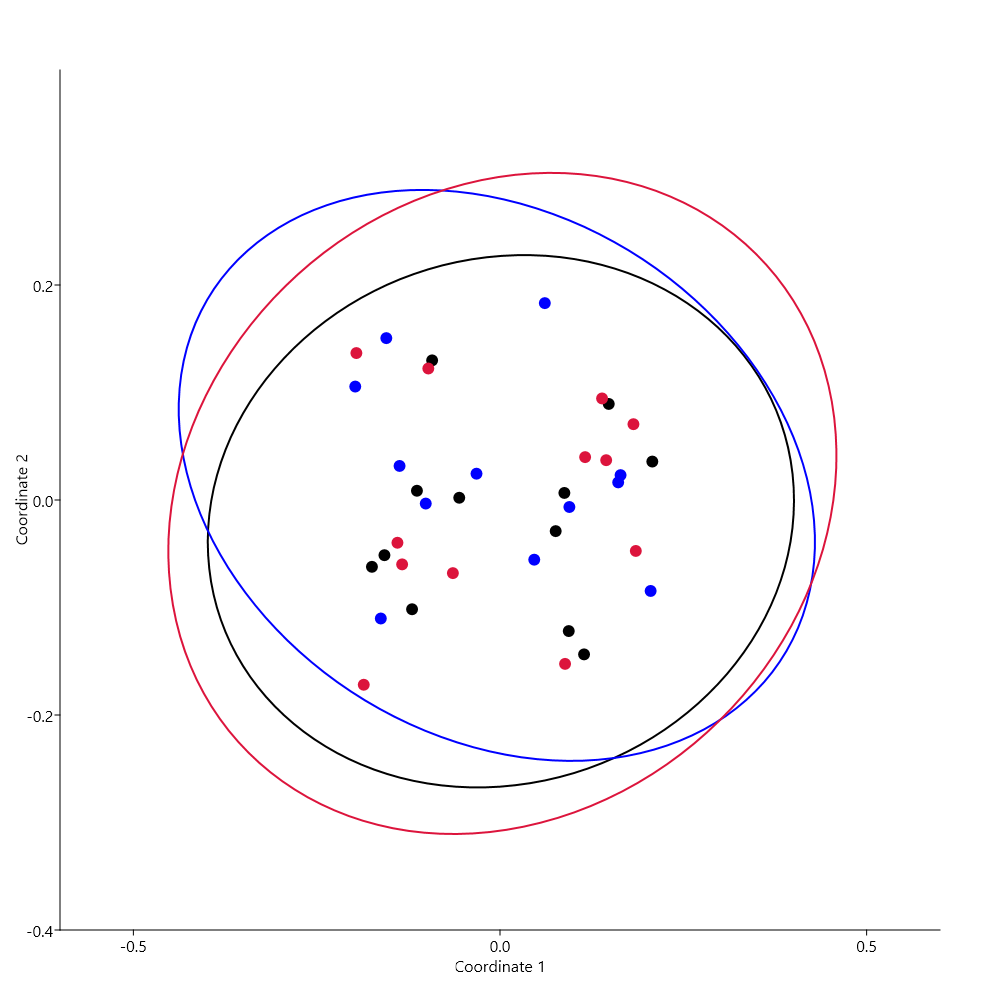
Appendix C**


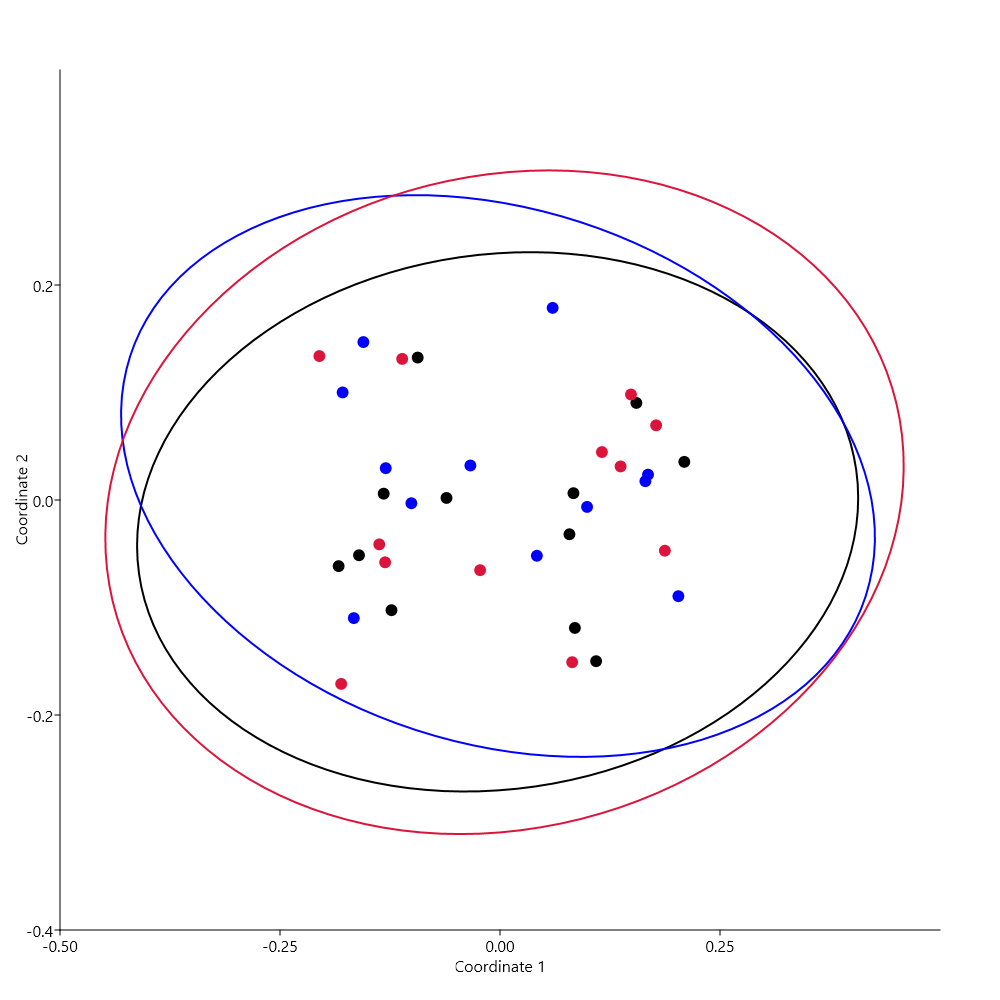


Fig. 1C ANOSIM plot of the different families per treatment (aquatic macro invertebrate data)

Fig. 2C ANOSIM plot of the different genera per treatment (aquatic macro invertebrate data)


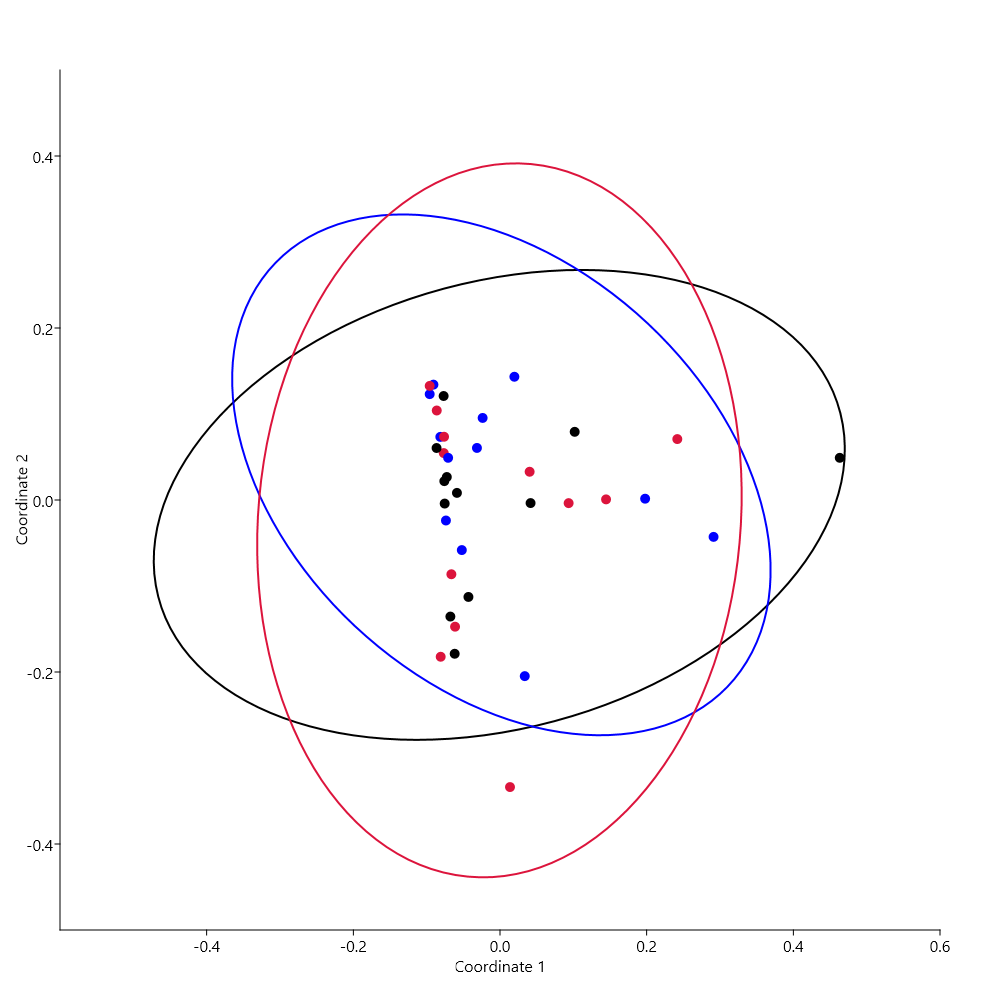


Fig. 3C ANOSIM plot of the different orders per treatment (aquatic macro invertebrate data)


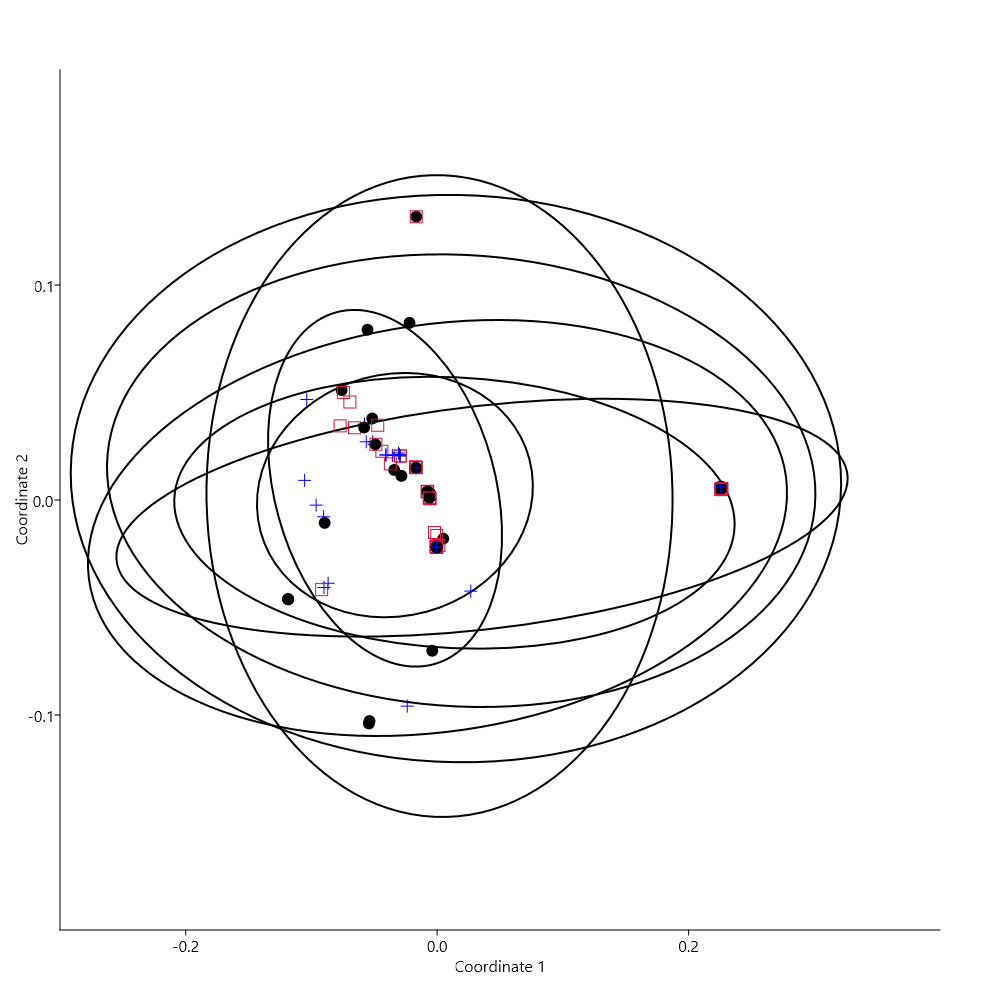


Fig. 4C ANOSIM plot of the different order per treatment (emergence data)

Appendix D

**Table 1** Species identified from aquatic macroinvertebrate sample collection

| **Order** | **Family** | **Genus** | **Species** | **Binomial name** |
| --- | --- | --- | --- | --- |
| Aranea | Cybaeidae | Argyroneta | aquatica | *Argyroneta aquatica* |
| Aranea | Lycosidae | Arctosa | leopardus | *Arctosa leopardus* |
| Aranea | Lycosidae | Pirata | piraticus | *Pirata piraticus* |
| Aranea | Lyniphyiidae | Lyniphiidae | NA | *Lyniphiidae sp.* |
| Aranea | Pisauridae | Dolomedes | fimbriatus | *Dolomedes fimbriatus* |
| Aranea | Tetrachnatidae | Tetrachnagnatha | extensa | *Tetrachnagnatha extensa* |
| Coleoptera | Dytiscidae | Colymbetes | fuscus | *Colymbetes fuscus* |
| Coleoptera | Dytiscidae | Agabus | bipistulatus | *Agabus bipistulatus* |
| Coleoptera | Dytiscidae | Hydroporinae | NA | *Hydroporinae sp.* |
| Coleoptera | Dytiscidae | Laccophilus | minutus | *Laccophilus minutus* |
| Coleoptera | Dytiscidae | Hygrotus | versicolor | *Hygrotus versicolor* |
| Coleoptera | Dytiscidae | Hygrotus | flavicollis | *Hygrotus flavicollis* |
| Coleoptera | Dytiscidae | Hydroglyphus | pictus | *Hydroglyphus pictus* |
| Coleoptera | Dytiscidae | Graptodytes | pictus | *Graptodytes pictus* |
| Coleoptera | Gyrinidae | Gyrinidae | NA | *Gyrinidae sp.* |
| Coleoptera | Hydrophilidae | Hydroporus | planus | *Hydroporus planus* |
| Coleoptera | Noteridae | Noterus | crassicornus c.f clavicornis | *Noterus crassicornus c.f clavicornis* |
| Coleoptera | Hydrophilidae | Laccobius | NA | *Laccobius sp.* |
| Coleoptera | Haliplidae | Haliplus | NA | *Haliplus sp.* |
| Coleoptera | Dytiscidae | Hydroglyphus | pussilus | *Hydroglyphus pussilus* |
| Coleoptera | Hydrophilidae | Enochrus | melanochephalus | *Enochrus melanochephalus* |
| Coleoptera | Haliplidae | Peltodytes | Caesus | *Peltodytes Caesus* |
| Coleoptera | Dytiscidae | Hydrobius | Fuscipes | *Hydrobius Fuscipes* |
| Coleoptera | Dytiscidae | Marginatis | NA | *Marginatis sp.* |
| Coleoptera | Dytiscidae | NA | NA | *NA* |
| Coleoptera | Hydrophilidae | Helophorus | sp. | *Helophorus sp.* |
| Coleoptera | Scirtidae | NA | NA | *NA* |
| Hemiptera | Gerridae | Gerris | odontogaster | *Gerris odontogaster* |
| Hemiptera | Naucoridae | Ilyocorus | cimicoides | *Ilyocorus cimicoides* |
| Hemiptera | Nepidae | Nepa | cinerea | *Nepa cinerea* |
| Hemiptera | Notonectidae | Notonecta | sp. | *Notonecta sp.* |
| Hemiptera | Pleidae | Plea | minutissima | *Plea minutissima* |
| Hemiptera | Gerridae | Gerris | thoracicus | *Gerris thoracicus* |
| Hemiptera | Corixidae | Sigara | NA | *Sigara sp.* |
| Hemiptera | Veliidae | Microvelia | reticulata | *Microvelia reticulata* |
| Hemiptera | Hebridae | Hebrus | pussilus | *Hebrus pussilus* |
| Odonata | Ceonagrionidae | Ishnura | elegans | *Ishnura elegans* |
| Megaloptera | Sialidae | Sialis | lutaria | *Sialis lutaria* |

**Table 2** Species identified from emergent macroinvertebrate sample collection

| Order | Family | Subfamily |
| --- | --- | --- |
| Coleoptera | Noteridae | NA |
| Diptera | Chaoboridae | NA |
| Diptera | Chironomidae | Orthocladinae |
| Diptera | Chironomidae | Tanypodinae |
| Diptera | Chironomidae | Chironominae |
| Diptera | Dixidae | NA |
| Diptera | Dolichopodidae | NA |
| Diptera | Empididae | NA |
| Diptera | Ephydridae | NA |
| Diptera | Muscidae | NA |
| Diptera | Simuliidae | NA |
| Diptera | Tipulidae | Tipula |
| Odonata | Ischnura | NA |
| Megaloptera | Sialidae | NA |

**Appendix E**

**Microcosm experiment**

**Methods**

To analyse the potential effect of bioaccumulation on predators of immature mosquitoes, a microcosm experiment was run. The goal was to demonstrate causality between predator death and Bti ingestion through exposed larvae. An *Ischnura elegans* (hereafter *I. elegans*) was kept in water of its natural habitat but was given exclusively Bti-exposed mosquito larvae. The predator, alongside the water, was gathered from a ditch at the Living Lab of Leiden University. The I. elegans was held in a glass jars (Ø 84 mm) containing 750 ml water from the Living Lab. It also contained a wooden stick to simulate a water plant for clinging. One *I. elegans* organism was held per container. The predator was fed 10 Bti-exposed mosquito larvae daily. Predator death and feeding behaviour was noted at 24h, 48h, and 72h. The control treatment was held under the same conditions to the setup described above, but mosquito larvae were not exposed to Bti. There were 12 replicates, so a total of 24 jars were used.

**Results**

When looking at the indirect effects on mosquito larvae predators (*Ischnura elegans*) through ingestion of exposed larvae, no significant effects were found on mortality over a period of three days (Df = 35, t = 1.78, p = 0.08). Within the microcosm, a significant difference was found between the control and Bti exposed predators in terms of mosquito larvae eaten (Df = 35, t = 2.82, p = 0.003). The control ate an average of 2.5 larvae whilst the Bti treatment ate 1.5 larvae.
